# Human and Porcine Hepatitis E Virus Strains, United Kingdom

**DOI:** 10.3201/eid1005.030908

**Published:** 2004-05

**Authors:** Malcolm Banks, Richard Bendall, Sylvia Grierson, Graham Heath, Jonathon Mitchell, Harry Dalton

**Affiliations:** * Veterinary Laboratories Agency, New Haw, Addlestone, Surrey, United Kingdom; † Royal Cornwall Hospital, Treliske, Truro, Cornwall, United Kingdom; ‡ Kings College Hospital, Denmark Hill, London, United Kingdom

**Keywords:** Hepatitis E, Zoonosis, Case history, Phylogenetics

## Abstract

We describe a case of acquired infection of a strain of hepatitis E virus (HEV)with a 100% amino acid identity to the analogous region in strains of HEV circulating in a United Kingdom pig herd. This case further supports the theory that autochthonous HEV infection in industrialized countries is zoonotic.

Hepatitis E virus (HEV) is a common cause of acute hepatitis in many developing countries (*1*). HEV is often waterborne and causes an illness similar to hepatitis A. During the icteric phase, patients may be viremic (*2*). In the last few years, sporadic cases of hepatitis E have been reported in industrialized countries in the absence of foreign travel or other known risk factors. Nucleotide sequence analysis of some of these viruses has indicated a high degree of sequence identity with the sequences of HEV detected in pigs (*3*–*5*) and in pig meat purchased in retail outlets in Japan (*6*). More recently, the most direct evidence of zoonotic transmission yet available was described in a case study from Japan. In this case, members of a family group were infected following consumption of uncooked Sika deer meat. The virus identified from the patients was shown to be identical to that recovered from uneaten quantities of meat from the same deer (*7*). In the United Kingdom, autochthonously acquired hepatitis E has been reported rarely and a short (98-bp) section of the virus genome has been sequenced from one such patient (*8*). Serologic evidence of HEV infection has been detected in U.K. pigs in a preliminary study (*9*) In two pigs from separate farms, an HEV genome was detected by reverse transcription–polymerase chain reaction (RT-PCR) and sequenced (strains 1-40 and 14-P354). These sequences identified the porcine strains as nearly identical to the 98-bp U.K. human HEV sequence in HEV genotype III (*9*). Further study is underway to determine the prevalence and strain diversity of HEV infection in U.K. pigs.

## The Study

In June 2000, a 58-year-old woman, who worked as a shop assistant, was seen at a rapid access “jaundice hotline” clinic with a 5-day history of myalgia and jaundice. She had not traveled outside the United Kingdom for 10 years and had no contact with farm or domestic animals. She was not a vegetarian and, although she admitted to eating raw sausage and bacon in the past, she claimed not to have done so in the 3 months before her illness. Examination confirmed jaundice and tenderness in the right upper quadrant. Her liver function tests showed elevated levels of bilirubin,46 μmol/L; alanine aminotransferase, 2,421 IU/L; and alkaline phosphatase, 200 IU/L. Blood drawn at this time was positive for anti-HEV immunoglobulin (Ig) M; no serologic evidence indicated active infection with Epstein-Barr virus, cytomegalovirus, or hepatitis A, B, or C. Results of a liver ultrasound were normal, and serologic evidence did not indicate autoimmune or metabolic liver disease. One month later, she felt better, was no longer jaundiced, and results of her liver function tests were generally normal. A diagnosis of acute hepatitis E was made.

Total RNA was extracted in Trizol (Sigma, Poole, UK) from a blood sample drawn from the patient at the time she sought treatment. Template cDNA from the blood extraction was prepared by a reverse transcriptase step, according to standard protocols. A nested PCR was used to detect HEV from the prepared cDNA samples. This assay used degenerate oligonucleotide primers to amplify a 348-bp fragment of open reading frame 2 (ORF2) of HEV as described by Meng et al. (1997) (*10*).

Cycling parameters were the same as those described by Meng et al. (*10*), except for annealing temperatures of 60°C and 55°C for external and internal primer pairs, respectively, and an extension time of 1 min. RT-PCR products were subjected to electrophoresis on a 2% agarose gel containing ethidium bromide and visualized under UV light.

Amplicons of correct size were excised from 2% agarose gels, purified with QIAquick Gel Extraction Kit (Qiagen Ltd., Crawley, UK), and sequenced with Big Dye Terminator Cycle Sequencing Ready Reaction (Applied Biosystems, Warrington, UK). Sequences comprising 304 bp of HEV ORF2 were assembled by SEQMAN (DNASTAR) and phylogenetic analysis was performed using PHYLIP (ver 3.6) with NEIGHBOR. Bootstrap confidence values were calculated by using SEQBOOT and CONSENSE (available from http://evolution.genetics.washington.edu/phylip.html).

**Figure 1:**
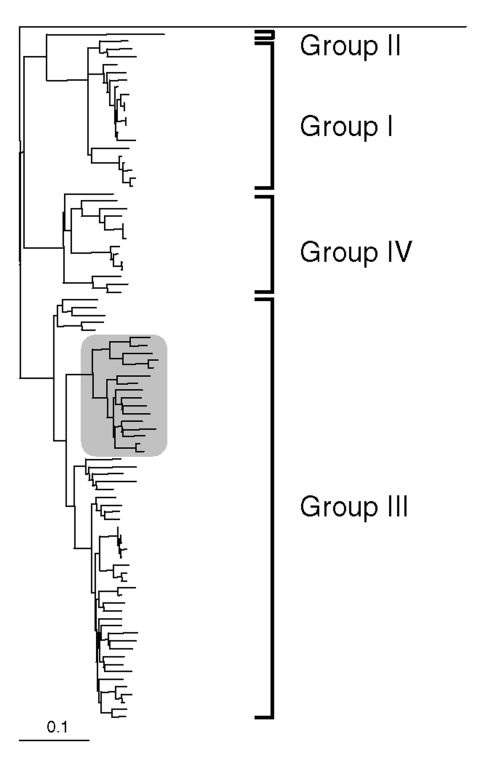
Outline of dendrogram of selected partial nucleotide sequences of ORF-2 region of swine and human hepatitis E virus (HEV) isolates (300 nt). Avian hepatitis E virus (AY043166) was chosen as an outgroup for these analyses. Genotypic groupings are indicated. Clustering of the human UK HEV isolate and closely related sequences is highlighted within shaded area (Figure 2).

**Figure 2:**
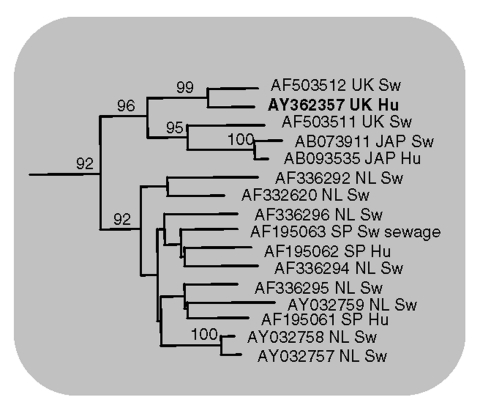
Human United Kingdom isolate (AY362357) is shown in bold and compared with closely related swine and human hepatitis E virus isolates (GenBank accession no., country of origin, and host are indicated). Bootstrap values greater than 70% are considered significant and are indicated.

Analysis of the 304-bp nucleotide sequence obtained between the primer sequences showed 16 and 32 nucleotide differences from the two U.K. pig strains 1-40 and 14-P354, respectively. However, all of these differences were silent when the gene sequence was translated into amino acids; the putative amino acid sequence of the virus detected in the patient had 100% identity with that of the two U.K. pig strains. Based on the nucleotide data the phylogeny of the three strains was determined (Figures 1, 2), showing a very close relationship between the three strains in the genotype III lineage. In Japan, high sequence similarities have been demonstrated between human and pig HEV strains within genotype III and genotype IV (*5*).

## Conclusions

HEV was discovered in pigs as recently as 1997 (*10*), despite evidence from several countries that it had been present since at least the early 1990s. The lack of recognized clinical disease in pigs (*11*) was undoubtedly a factor in this late discovery. The similarity of the human strain detected in this case to the two U.K. pig strains is suggestive of zoonotic transmission, as is the finding of higher prevalence of anti-HEV antibodies in those who work with pigs (*12*) and the observation that in 9 of 10 hepatitis E patients in Japan, the disease developed within 2–8 weeks of consumption of grilled or undercooked pork (*6*). Consideration is being given within the U.K. Department for Environment, Food and Rural Affairs to research proposals to gain better understanding of HEV in the U.K. pig herd. Screening patients who present with acute hepatitis for evidence of HEV infection is a simple way to improve the assessment of human HEV infection. Rapid access jaundice hotline clinics (*13*) are an ideal venue for such testing because patients may present while still viremic, allowing molecular characterization of infecting viruses as in this case. Work is currently in progress to assess the incidence of hepatitis E in a cohort of over 650 acutely jaundiced patients who have contacted the jaundice hotline in the past 5 years.
